# 

**Published:** 1852-02

**Authors:** 


No. IX. FEBRUARY. Vol. VII.

### BUFFALO
### MEDICAL JOURNAL
AND
### MONTHLY REVIEW
OF
### MEDICAL AND SURGICAL SCIENCE.

EDITED BY AUSTIN FLINT, M. D.

BUFFALO:
PUBLISHED BY JEWETT, THOMAS & CO.
Commercial Advertiser Buildings.
1852

*Price $2.50 per annum . . . in Advance.*

